# Individual Differences in Social Behavior and Cortical Vasopressin Receptor: Genetics, Epigenetics, and Evolution

**DOI:** 10.3389/fnins.2017.00537

**Published:** 2017-10-04

**Authors:** Steven M. Phelps, Mariam Okhovat, Alejandro Berrio

**Affiliations:** ^1^Department of Integrative Biology, University of Texas at Austin, Austin, TX, United States; ^2^Department of Medicine, Oregon Health and Science University, Portland, OR, United States; ^3^Department of Biology, Duke University, Durham, NC, United States

**Keywords:** cognitive ecology, balancing selection, enhancer elements, single nucleotide polymorphism, *Microtus ochrogaster*, neuroendocrinology, monogamy

## Abstract

Social behavior is among the most complex and variable of traits. Despite its diversity, we know little about how genetic and developmental factors interact to shape natural variation in social behavior. This review surveys recent work on individual differences in the expression of the vasopressin 1a receptor (V1aR), a major regulator of social behavior, in the neocortex of the socially monogamous prairie vole. V1aR exhibits profound variation in the retrosplenial cortex (RSC), a region critical to spatial and contextual memory. RSC-V1aR abundance is associated with patterns of male space-use and sexual fidelity in the field: males with high RSC-V1aR show high spatial and sexual fidelity to partners, while low RSC-V1aR males are significantly more likely to mate outside the pair-bond. Individual differences in RSC-V1aR are predicted by a set of linked single nucleotide polymorphisms within the *avpr1a* locus. These alternative alleles have been actively maintained by selection, suggesting that the brain differences represent a balanced polymorphism. Lastly, the alleles occur within regulatory sequences, and result in differential sensitivity to environmental perturbation. Together the data provide insight into how genetic, epigenetic and evolutionary forces interact to shape the social brain.

Individual differences in social behavior are remarkably common. Male lizards vary dramatically in their display colors and aggressive behaviors (Sinervo and Lively, 1996). Male sunfish may differ profoundly in their parental care (Gross, 1991), while bluehead wrasses can shift body color, behavior, and even sex in response to social environments (Semsar and Godwin, 2004). Indeed, evolutionary theory has long known that the fitness value of specific behavioral traits may depend on the frequency of such traits in the population, or on the population density of conspecifics (Maynard-Smith and Price, 1973). Similar forces have been hypothesized to shape individual differences in human personality (Keller and Miller, 2006; Penke et al., 2007), resilience to developmental trauma (Boyce and Ellis, 2005), and even the variety of human faces (Sheehan and Nachman, 2014). Understanding the genetic and epigenetic factors that shape individual differences in social behavior is thus of fundamental importance to both our basic understanding of behavior, and to our understanding of natural variation related to health and disease.

Behavioral neuroscience is often focused on model species in which genetic diversity has been intentionally purged. This has the advantage of minimizing variation that could confound the study of species-specific traits, and this strategy has enabled substantial insights into the role of developmental factors in shaping adult behavior. Intrauterine environments (Ryan and Vandenbergh, 2002), parental care (Weaver et al., 2004), and environmental complexity (van Praag et al., 2000), for example, all have profound influences on the development of brain and behavior. The decision to study genetically similar individuals, however, precludes studying genetic variation or how it interacts with developmental environments to shape natural behavior.

Non-traditional model species offer a variety of strengths that complement traditional foci of behavioral neuroscience (Phelps, 2010; Taborsky et al., 2015). For example, by studying species in which genetic diversity has been actively retained by derivation from wild stock, it is possible to examine how genetic variation contributes to brain and behavior. In addition, species may be chosen that exhibit interesting social phenotypes not exhibited by traditional model systems. Among mammals, recent examples include the study of pair-bonds (Young and Wang, 2004; Ophir et al., 2007), non-sexual bonds (Beery and Zucker, 2010), elaborate vocalizations (Blondel and Phelps, 2009; Crino et al., 2010), and the elaboration of paternal care (Bendesky et al., 2017). Work on non-traditional rodents and primates, moreover, can employ many of the technologies developed for common mammalian models (e.g., Lim et al., 2004). These attributes make them powerful supplements to common approaches in social neuroscience.

In the current paper, we offer a detailed review of our work on individual differences in the vasopressin system of prairie voles, a socially monogamous rodent that has become a powerful model for the study of attachment. We focus more specifically on cortical differences in the abundance in the vasopressin 1a receptor, the predominant form in the brain. Our focus, the retrosplenial cortex (RSC), is a brain region critical to spatial and contextual memory, and an increasing focus of research in both humans and rodents (Harker and Whishaw, 2002; Vann et al., 2009; Kingsbury et al., 2012; Ranganath and Ritchey, 2012; Cowansage et al., 2014; Todd and Bucci, 2015). The expression of V1aR in the RSC is profoundly variable among individual prairie voles, and has been linked to both spatial behavior and sexual fidelity in the wild (Figure 1). We begin by introducing prairie voles as models in social neuroscience and neuroendocrinology.

**Figure 1:**
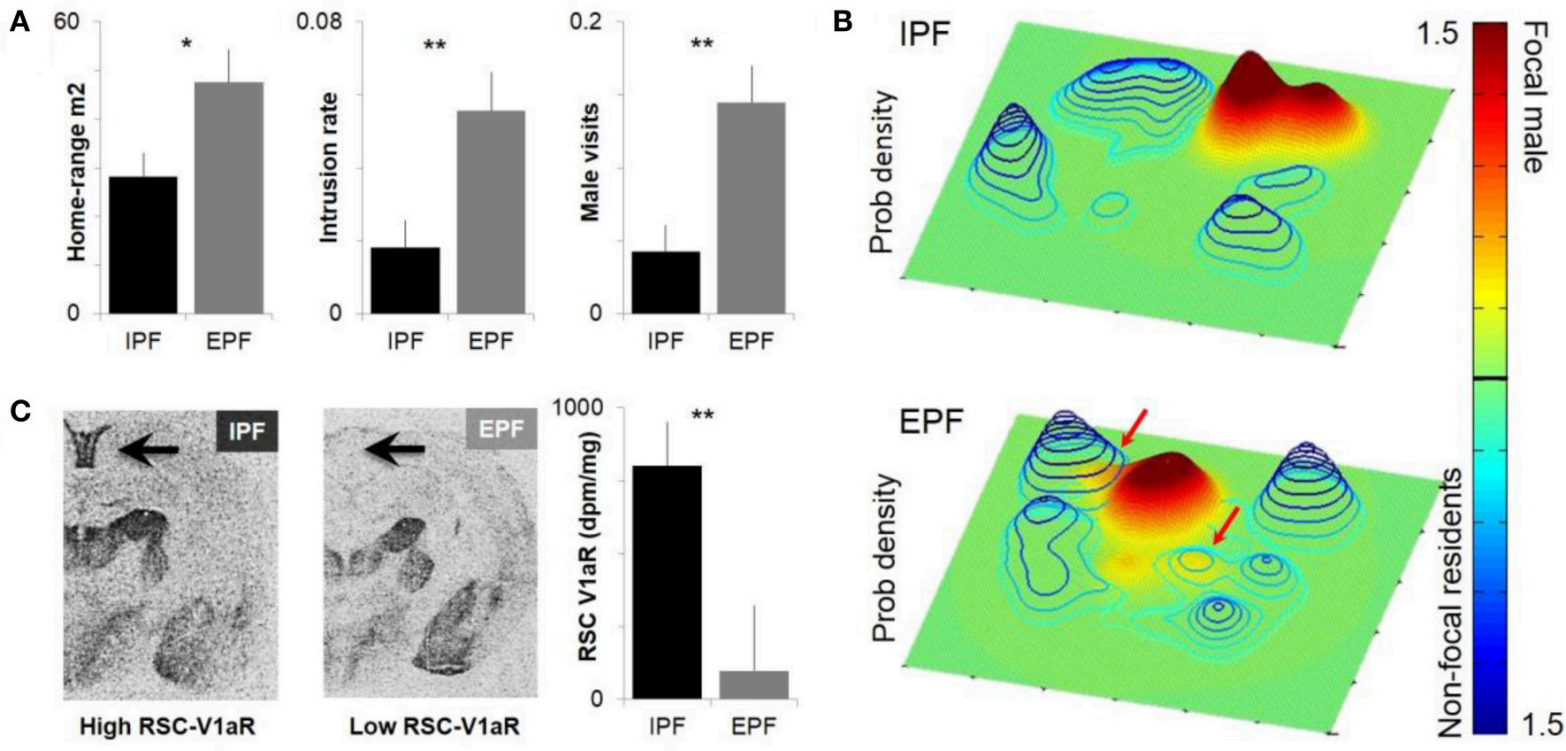
Individual differences in male space-use and sexual fidelity are predicted by RSC-V1aR abundance. **(A)** Males who sired young only within a pair (intrapair fertilization, IPF) and males who sired at least one embryo with a non-partner (extrapair fertilization, EPF) differ in homerange size, the rate of intrusions onto resident male territories, and the rate at which their own core homeranges were visits by other males. **(B)** Sample probability density estimates of paired males within a common enclosure. Focal males are shown on a green-red scale and a solid surface, non-focal residents in blue wired surface. X and Y axes correspond to dimensions of the outdoor enclosure. Top panel shows an IPF male that does not intrude on the territories of other bonded males; bottom panel depicts an EPF male who intrudes on two other male territories. Colorbar depicts probability density (0–1.5 × 10e-3) that a focal male or non-focal resident will be at a given point in space. **(C)** IPF and EPF males differ in the abundance of V1aR in the RSC, which predicts individual differences in space use. ^*^P < 0.05, ^**^P < 0.01. Modified with permission from Okhovat et al. (2015).

## Prairie voles as models of monogamy

The prairie vole, *Microtus ochrogaster*, is a small North American rodent that lives in grasslands. It is known both for its wild fluctuations in population density and for its ability to form enduring pair-bonds (Thomas and Birney, 1979; Carter et al., 1986, 1995; Getz et al., 1993, 2001; Pizzuto and Getz, 1998). Males and females live in pairs and share care of offspring. Roughly 25% of these young are sired outside the pair (Ophir et al., 2008b). Male pair-bonding is accompanied by a dramatic increase in aggression and a reduction in homerange. While paired males live as aggressive, territorial “residents,” up to 45% of males may live as unpaired, non-territorial “wanderers” (Getz et al., 1993; Solomon and Jacquot, 2002).

Space use is a critical component of variation both within and between these alternative male tactics. Residents with small, exclusive homeranges have high mating success with their respective partners (Ophir et al., 2008c; Phelps and Ophir, 2009). Residents with larger homeranges gain extra-pair fertilizations (EPFs) but are more often cuckolded. Wanderers have larger, less exclusive homeranges than residents, but only those with the largest homeranges obtain EPFs (Ophir et al., 2008c; Phelps and Ophir, 2009). Thus, for both residents and wanderers, larger and less exclusive homeranges translate into increases in extrapair paternity; only residents, however, face trade-offs between EPFs and IPFs. Space use differs between residents and wanderers, but it also predicts patterns of paternity within tactics.

## Vasopressin and mating sytem

Arginine-vasopressin (AVP) is a nine-amino acid peptide implicated in a wide variety of social behaviors. Among vertebrates, AVP and its homologs are commonly linked to aggression, courtship and other social behaviors (Goodson and Bass, 2001; Caldwell et al., 2008). Among mammals, neurons of the bed nucleus of the stria terminalis and the medial amygdala express AVP at higher levels in males than females (De Vries et al., 1994), a finding thought to contribute to the importance of the peptide to male social behavior (Cho et al., 1999; but see Bosch, 2013; Dumais and Veenema, 2016, for examples of vasopressin functions in female social behaviors). Although this neuropeptide is consistently implicated in social behavior, its effects can be highly species-specific. This specificity seems to emerge from species differences in the neural distribution of the vasopressin 1a receptor (V1aR). Prairie voles, for example, exhibit high V1aR in a reward region, the ventral pallidum, that influences pairbond formation (Winslow et al., 1993; Insel et al., 1994). Injection of a vasopressin antagonist into the ventral pallidum blocks pair-bonding in male prairie voles (Lim and Young, 2004). Remarkably, viral overexpression of pallidal V1aR enables normally promiscuous male meadow voles to form attachments (Lim et al., 2004).

Although the ventral pallidum causes species differences in pair-bond formation among voles, this mechanism does not seem to be general. We recently measured pallidal V1aR in seven species of *Peromyscus*, for example, and found it did not reliably predict mating system across deer mice (Turner et al., 2010). The consensus seems to be that the ability of vasopressin and its homologs to modulate social behavior is an ancient innovation common among vertebrates (Goodson, 2005; Ho et al., 2010; O'Connell and Hofmann, 2011, 2012). The effects of the hormone on a particular social behavior, however, can emerge in a variety of ways, presumably by acting anywhere in a series of connected brain regions important for social behavior (e.g., Goodson, 2005; O'Connell and Hofmann, 2011, 2012).

Although differences between monogamous and promiscuous vole species are shaped by pallidal V1aR, residents and wanderers have *identical* patterns of neural V1aR (Ophir et al., 2008c). The abundance of V1aR in the ventral pallidum is remarkably consistent across individual prairie voles, with the high levels needed for pair-bonding apparently fixed within the population (Phelps and Young, 2003). Given that bonded males have higher fitness, it seems likely that selection has cleared heritable variation in pallidal V1aR abundance (Phelps and Ophir, 2009). Differences between resident and wandering males seem to represent differences in opportunity rather than neural V1aR abundance (Ophir et al., 2008c). Somewhat surprisingly, although there are no differences in V1aR between residents and wanderers, more subtle behavioral variation within each tactic is associated with the abundance of V1aR in the RSC (Ophir et al., 2008c; Figure 1).

To examine this relationship, we collared and radiotracked animals in the field, using the locations determined over the course of a few weeks to estimate the probability a given animal would be at a particular point in space (Ophir et al., 2008c; Okhovat et al., 2015; Figure 1). From these probability landscapes, we can estimate the core of an animal's homerange, and the extent to which the animal intrudes into the core homeranges of its neighbors. The data reveal that having low RSC-V1aR is associated with more territorial intrusion, increased rates of being intruded upon, and increased extra-pair paternity (Phelps and Ophir, 2009; Okhovat et al., 2015; Figure 1). [Interestingly, RSC-V1aR was not associated with female behavior (Zheng et al., 2013)]. Together these data suggest that vasopressin function shapes individual differences in memory, space-use and sexual fidelity in the field. Given the prominent role of the RSC in spatial memory, we hypothesize that males with low RSC-V1aR are less adept at remembering the spatial location of social interactions. In this scenario, low RSC-V1aR males intrude more because they are less able to recall the details of a punitive encounter with a resident male; males with high RSC-V1aR, in contrast, seem to avoid intruding on male territories, and so are better equipped to guard their mates. An alternative (but not mutually exclusive) hypothesis is that RSC-V1aR influences space use and sexual fidelity by shaping the strength of a bond, or by promoting a male's ability to keep track of his mate. These alternatives have yet to be tested.

## Genetic variation at the Avpr1a locus predicts RSC-V1aR expression

Having identified profound individual differences in cortical V1aR (Phelps and Young, 2003), and linked them to individual differences in behavior (Ophir et al., 2008c; Phelps and Ophir, 2009), we next asked whether individual differences in RSC-V1aR abundance were genetic, epigenetic, or both. From our field data, there were two plausible explanations: that differences in behavioral experiences somehow drove the individual differences in V1aR, or that V1aR in the RSC preceded and perhaps caused the behavioral differences in space-use and fidelity. If RSC-V1aR variation caused behavioral differences, what was the origin of the cortical variation?

A variety of findings suggested that RSC-V1aR was not caused by the experience of intra-pair or extra-pair paternity, but was some complex combination of genetic and developmental regulation of the *avpr1a* locus. First, there are no sex differences in RSC-V1aR abundance, nor are there any differences between paired and single animals (Phelps and Young, 2003; Ophir et al., 2008c), suggesting that neither sex steroids nor mating experience influenced expression. Moreover, work by Hammock and Young (2005) bred lines of prairie voles with long or short microsatellite lengths in the *avpr1a* promoter, and found that they differed substantially in RSC-V1aR abundance. This demonstrates that *cis*-acting sequence variation contributes to RSC-V1aR. The story became more complicated, however, because neither Hammock et al. (2005), nor our own lab (Ophir et al., 2008a) found microsatellite length to predict RSC-V1aR in outbred animals. Our hypothesis was that the *avpr1a* microsatellite is not causal, but that it was imperfectly linked to neighboring single-nucleotide polymorphisms (SNPs) that are responsible for individual differences. Studies that bred for long or short microsatellites would also select for different frequencies of any linked SNPs.

**Figure 2:**
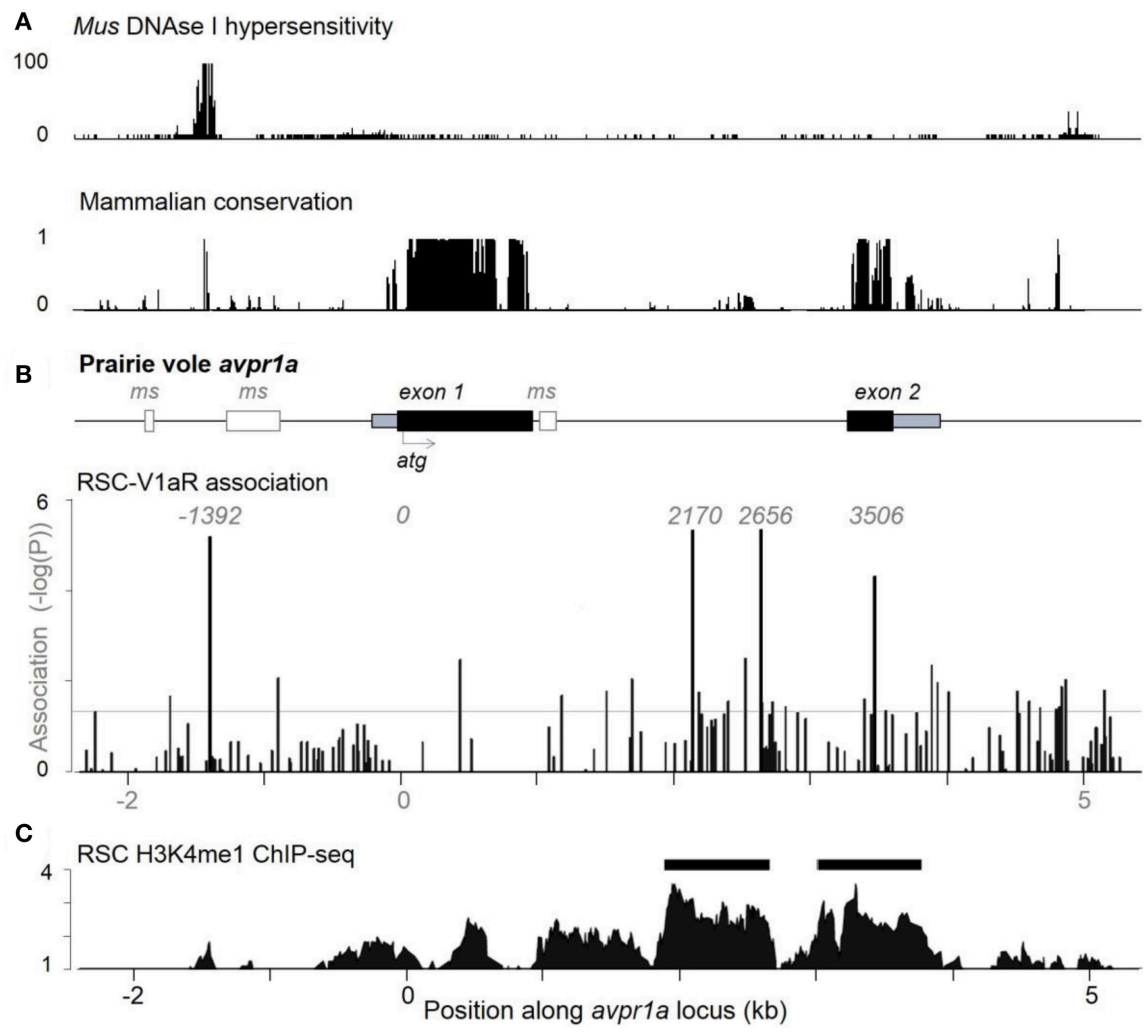
Individual differences in RSC-V1aR abundance are well predicted by 4 linked single nucleotide polymorphisms at the *avpr1a* locus. **(A)** ENCODE data on DNAse hypersensivity (top) from the cortex of adult *Mus musculus* (top), and conservation of corresponding sequences across mammals (below). **(B)** The structure of the prairie vole avpr1a locus includes two exons (UTRs in gray, CDS in black), and three microsatellite sequences (ms, white). The microsatellite upstream of the first exon has been the subject of numerous studies. Below, vertical bars represent the strength of association (-logP) between each identified SNP and RSC-V1aR abundance. Four strongly linked SNPs (positions -1392, 2170, 2656, 3506) were highly associated with RSC-V1aR and survived multiple comparison corrections. Horizontal bar corresponds to uncorrected α (*P* = 0.05). **(C)** Chromatin immunoprecipitation sequencing (ChIP-seq) targeting the enhancer marker H3K4me1 reveals significant enrichment (compared to input DNA controls) within the intron, as well as within the second exon. Horizontal scale depicts position in kilobases (kb) of *avpr1a* locus, aligned to all panels in figure. Data used in association analyses included both males and females. Modified with permission from Okhovat et al. (2015).

To test the hypothesis that SNPs were shaping RSC-V1aR abundance, we looked at natural variation in RSC-V1aR and sequence variants from a large population of lab-reared and wild-caught prairie voles across ~8 kb of the *avpr1a* locus (Figure 2). We found 151 SNPs overall (Okhovat et al., 2015). None of these SNPs predicted V1aR in brain regions implicated in bonding and aggression (ventral pallidum or lateral septum). However, we found four tightly linked SNPs predicted RSC-V1aR. These four SNPs were found upstream of the first exon (SNP -1392), within the intron (SNP 2170 and 2676) and in the second exon (SNP 3506; all SNPs are numbered with respect to translation start site; Figure 2B). We refer to the set of SNPs that correspond to high RSC-V1aR as the HI allele, and the opposite set of SNPs as the LO allele. We replicated this association on a third population of wild-derived animals, crossing parents heterozygous for the alleles to produce HI/HI and LO/LO homozygotes in the same litter (Okhovat et al., 2015). We found that the HI and LO alleles were strong, replicable, and robust predictors of not only RSC-V1aR, but also *avpr1a* transcript abundance, suggesting that these predictive SNPs affect *avpr1a cis*-regulation.

When located within regulatory regions, SNPs can alter gene expression by changing the epigenetic properties of the locus. Remarkably, all four RSC-associated *avpr1a* SNPs co-localized with markers of gene regulation. SNP -1392 was within an deoxyribonuclease I (DNAse I) hypersensitive site, a marker of open chromatin; moreover, this open chromatin was centered on a highly conserved binding site for the transcription factor CTCF, a factor known to shape gene regulation through its contributions to chromatin looping (Phillips and Corces, 2009, Figure 2A). Distal regulatory sequences that interact directly with promoters to regulate transcription are known as enhancers, and can be identified through their characteristic histone modifications (Heintzman et al., 2009). One such mark is the monomethylation of lysine 4 in histone 3 (H3K4me1), which marks both active and poised enhancers (Heintzman et al., 2009). We performed ChIP-seq on the RSC of prairie voles, and found that one such enhancer site was located in the *avpr1a* intron and overlapped with both SNP 2170 and SNP 2676 (Figure 2C). There was also a second putative enhancer that overlapped with the second *avpr1a* exon and SNP 3506 in the HI/LO alleles.

Interestingly, SNP 2170 is a T/G polymorphism that alters the presence/absence of a CpG site located within a putative intron enhancer. This site is also weakly linked to additional polymorphic CpG sites (polyCpG) within the same enhancer, leading to significant HI and LO allelic differences in CpG availability; the LO allele, which is associated with lower RSC-V1aR, has significantly more CpG sites in the putative intron enhancer compared to the HI allele (Okhovat et al., 2015, 2017b). CpG sites are the main targets for DNA methylation—a well-known epigenetic modification that can regulate gene expression—therefore, we hypothesized that *avpr1a* genotype differences in enhancer CpG could lead to differences in enhancer methylation and *avpr1a* expression in the RSC.

We found that both lab-reared and wild-caught showed significantly different levels of DNA methylation in the intron enhancer (Okhovat et al., 2015, 2017b). There was also a negative correlation between overall enhancer methylation and *avpr1a* transcription (Okhovat et al., 2015, 2017a), suggesting that enhancer methylation lowers RSC-V1aR by reducing *avpr1a* transcription, consistent with commonly reported silencing effects of DNA methylation (Nan et al., 1998). While enhancer methylation predicted individual differences in RSC-V1aR, methylation of the *avpr1a* promoter did not (Okhovat et al., 2017a). Although promoter methylation is generally silencing (Bird and Wolffe, 1999), our data indicate that the *avpr1a* promoter is generally un-methylated, whether the locus is active or not. This finding is in line with recent studies that suggest promoters are often unmethylated, even in cell types in which they are not expressed—thus a lack of methylation is necessary but not sufficient for gene expression (Rollins et al., 2006; Lister et al., 2013). Methylation and sequence variation in regulatory elements outside of the promoter area—especially within enhancer sequences—may be better predictors of expression.

A detailed analysis of HI and LO allele sequences suggested at least two mechanisms by which sequence variation and epigenetic mechanisms might interact at the *avpr1a* enhancer. First, allelic differences in CpG abundance and overall enhancer methylation could account for differences in expression via allele-biased recruitment of repressive methyl-binding proteins—such as MeCP2 (Bird, 2002). Alternatively, binding of transcription factors may be influenced by sequence changes generated by SNP 2170. Based on published position weight matrices, some transcription factors, including GATA2—which is expressed in the mouse RSC—bind preferably to the LO allele (Okhovat et al., 2017b). Therefore, differential binding of transcription factors due to both genetic and epigenetic variation at the intron enhancer may drive allele-biased changes in RSC-V1aR abundance. While further research is required to elucidate the exact molecular consequences of sequence variation in the intron enhancer, these findings provide promising explanations for the variation observed in RSC-V1aR.

## Selection maintains allelic variation related to RSC-V1aR abundance

Although individuals can vary tremendously in social behavior, as well as in gene expression and brain function, we know relatively little about how DNA sequence variation contributes to meaningful differences in brain and behavior. We have reviewed data showing that individual differences in RSC-V1aR predict behavior of male prairie voles in the field, and that these brain differences are due at least in part to genetic variation at the *avpr1a* locus. Here, we examine whether there is evidence that natural selection has actively maintained variation in brain in behavior.

**Figure 3:**
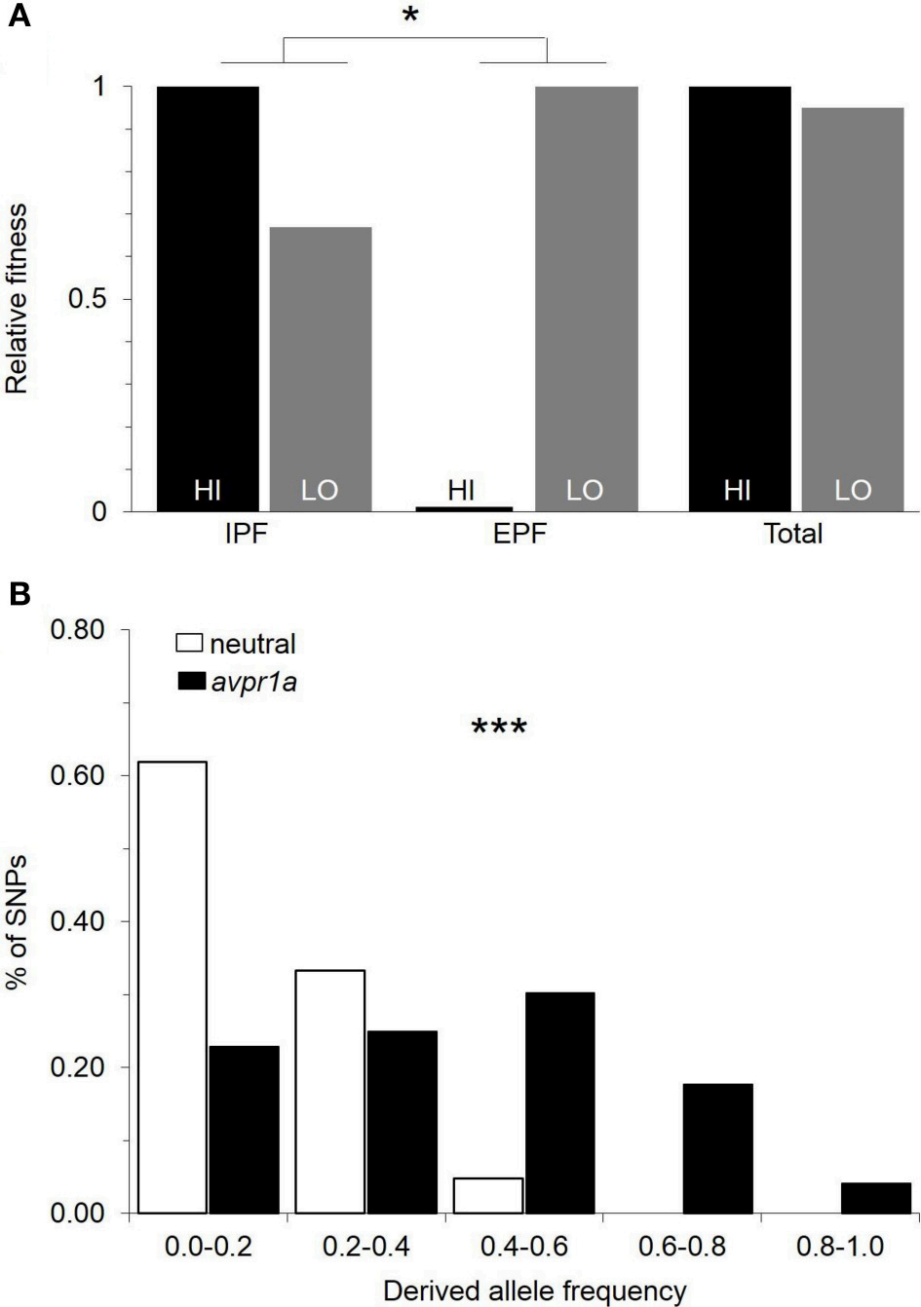
Natural selection maintains variation in RSC-V1aR abundance. **(A)** Relative fitness of HI and LO alleles measured in the context of intrapair (IPF) and extrapair (EPF) fertilization rates obtained by male prairie voles. Selection is measured by the difference in fitness of the two alleles. The differences in the direction and strength of selection in IPF and EPF contexts were tested with a permutation test. **(B)** Comparison of the frequency spectra of polymorphisms for *avpr1a* (black) and neutral loci (white) reveals a significant excess of intermediate frequency alleles in *avpr1a*. ^*^P < 0.05, ^***^P < 0.001. Modified with permission from Okhovat et al. (2015).

Our first analysis was to revisit data on paternity and fitness obtained from animals in the field (Figure 1). We asked whether there was a difference between paired and single males in their overall fitness, as measured by the number of pups that they sired in the field. We found that paired males sired more young (Ophir et al., 2008b), demonstrating that selection favors the capacity to form pair-bonds. However, we did not find a difference in the fitness of males who mated exclusively with a partner (IPF), and those who mated at least once outside a pairbond (EPF), suggesting that both faithful and unfaithful males do comparably well in the conditions we examined (Ophir et al., 2008c). We examined the relative fitness of HI and LO RSC alleles in our field study and found that they did not differ significantly in fitness (Figure 3A; Okhovat et al., 2015). However, when we examined how this fitness was obtained, we found that HI alleles were more fit than LO alleles in the context of intra-pair fertilizations, while LO alleles were more fit in the context of extra-pair fertilizations (Figure 3A; Okhovat et al., 2015). This is consistent with the view that the diversity of V1aR in the RSC represents a “balanced polymorphism” of the social brain, in which faithful and unfaithful male mating behaviors provide alternate but equivalent sources of evolutionary fitness.

While these results were encouraging, our field studies were a snapshot in time, tested under a single set of population densities and over a narrow range of conditions. We used evolutionary genetic approaches to test whether there was a history of selection actively maintaining variation at the *avpr1a* locus. A new mutation is, by definition, at low frequency within a population, and in the absence of selection, it is more likely to be lost than to drift to high frequency; thus most variable sites in the genome are at low frequencies (Hudson et al., 1987; Tajima, 1989). If selection is actively maintaining alternative forms of an allele, however, both forms tend to be at intermediate frequencies, and neighboring sites are also at higher frequencies than is characteristic of the genome as a whole (Hudson et al., 1987; Tajima, 1989). We compared the frequencies of mutations at the *avpr1a* locus to those in three putatively neutral genes (Okhovat et al., 2015), or across the entire genome (Berrío Escobar, 2017). We found that indeed, the *avpr1a* locus had higher frequencies of SNPs than was characteristic of the rest of the genome, suggesting that selection actively maintained this diversity (Figure 3B). Moreover, this signal was concentrated in the vicinity of the SNPs that defined the HI and LO alleles—a region of the *avpr1a* locus that did not predict expression in other brain regions (Okhovat et al., 2015; Berrío Escobar, 2017). Together these data suggest that RSC-V1aR diversity represents adaptive variation in brain, behavior, and cognition.

The high degree of linkage between the SNPs that defined HI and LO alleles seems unusual, because many intervening polymorphisms are unlinked to HI and LO alleles. We used permutation tests to ask whether these SNPs were significantly more linked than we would expect by chance (Berrío Escobar, 2017). We found that the SNPs were significantly more linked than predicted based on the distance between them—a pattern suggesting that the selection had favored specific combination of nucleotides across sites. Such epistasis across regulatory regions is poorly studied, but not without precedent. For example, recent data suggest that SNP-by-SNP interactions among non-coding elements play an important role in human disease (Dinu et al., 2012; Jamshidi et al., 2015). Such epistasis may reflect interactions among transcription factors that bind at different sites, contributions to chromatin looping and conformation, or any of the many other molecular changes needed to effectively coordinate transcription at a locus (e.g., Grubert et al., 2015). Whether the HI and LO SNPs interact remains to be determined, but our evidence of non-random linkage further suggests a causal role for these polymorphisms.

## Developmental variation at the *avpr1a* locus

Although SNPs in *avpr1a* regulatory sequences seem to have a major role in regulating RSC-V1aR abundance, a variety of data suggested that environmental factors may also be at play. For example, lab-reared voles had a stronger association between HI and LO alleles and RSC-V1aR abundance than did wild-caught prairie voles (Okhovat et al., 2015). This observation suggested that RSC-V1aR variation might also be shaped by the environmental variation that voles are naturally exposed to in the wild (e.g., population and resource fluctuations, Getz et al., 2001). In fact, previous work on prairie voles (Bales et al., 2007; Prounis et al., 2015) and rats (Francis et al., 2002) demonstrated that developmental manipulations can alter V1aR regulation in the RSC and other brain regions. While the exact molecular mechanisms for these neuronal changes are not known, environmentally induced changes in neuronal gene expression are often mediated by molecular epigenetic modifications, such as DNA methylation (Szyf and Bick, 2013). Given that HI and LO alleles differ in the abundance of CpG sites within the putative intron enhancer, and that the methylation of this enhancer is negatively associated with RSC-V1aR abundance (Okhovat et al., 2015, Figure 4A), we hypothesized that LO alleles may be more sensitive to developmental perturbations that influence CpG methylation.

**Figure 4:**
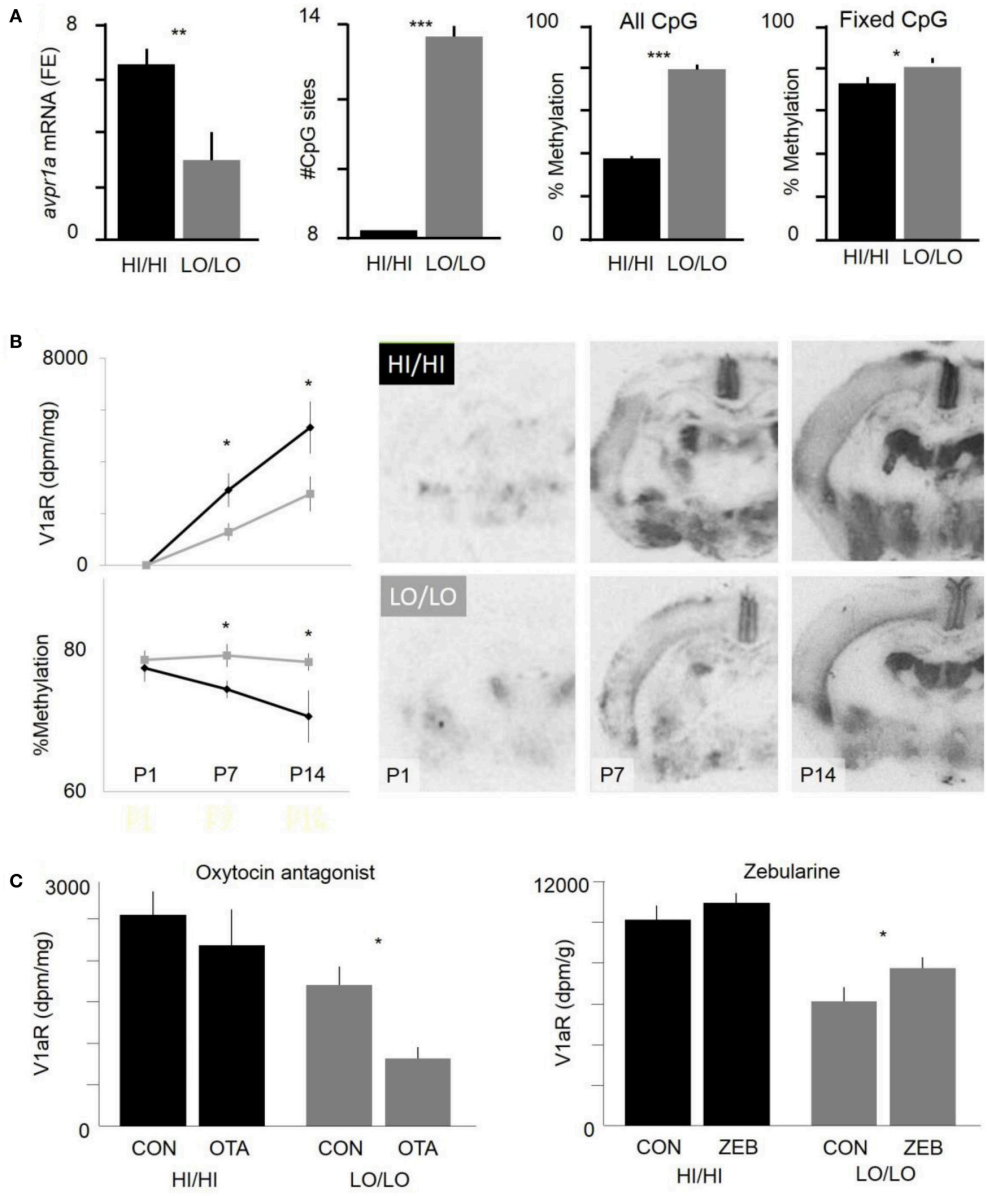
Interaction of genetic and epigenetic differences in cortical V1aR abundance. **(A)** Effects of HI/LO genotype on RSC *avpr1a* mRNA abundance (fold enrichment, far left), the number of CpG sites within the intron enhancer, the methylation status of the intron enhancer, and the methylation status of enhancer CpGs that lack sequence variation (“fixed CpGs”, far right). Graphs represent animals at weaning age (21d). **(B)** Ontogeny of RSC-V1aR abundance (top left) and methylation of fixed CpGs within the intron enhancer (bottom left). Images represent autoradiograms of brains with median RSC-V1aR abundance for each genotype and time point (postnatal days 1, 7, and 14). **(C)** Genotype-specific effects of neonatal (P1) manipulations of oxytocin antagonist (left) and a methylation inhibitor (right) on RSC-V1aR abundance. ^*^P < 0.05, ^**^P < 0.01, ^***^P < 0.001. Data included both male and female offspring. Modified with permission from Okhovat et al. (2015, 2017a).

Typically, rodent brains undergo periods of dramatic developmental change in gene expression and methylation; such critical periods are often highly responsive to environmental variation in parental care, diet, or stress (Roth and Sweatt, 2011). However, based on genetic makeup, individuals can vary in their sensitivity and response to these early developmental perturbations, a phenomenon known as gene-by-environment interactions (GxE). In prairie voles, neuronal V1aR abundance undergoes drastic changes postnatally (Wang et al., 1997). To begin to understand how genotype interacts with development, we examined the ontogeny of RSC-V1aR in HI/HI and LO/LO genotypes. We found that one-day-old HI/HI and LO/LO voles lacked RSC-V1aR (Okhovat et al., 2017a). However, significant genotype differences in RSC-V1aR quickly emerge during the first postnatal week (Figure 4B). Interestingly, genotype differences in *avpr1a* enhancer methylation also appear during this period, indicating that enhancer methylation may be involved in early-life regulation of RSC-V1aR (Okhovat et al., 2017a, Figure 4B).

To assess HI and LO differences in susceptibility to early-life perturbation, newborn pups were exposed to oxytocin receptor antagonist, a manipulation that is sometimes considered analogous to poor parenting, and that has been shown to alter adult RSC-V1aR of voles (Bales et al., 2007). This postnatal treatment reduced RSC-V1aR later at weaning age (21 days), demonstrating that *avpr1a* regulation is sensitive to early developmental and environmental perturbations (Okhovat et al., 2017a, Figure 4C). This sensitivity, however, was only detected in LO/LO pups, and not their HI/HI siblings. Similarly, we used a global inhibitor of methylation, zebularine (Cheng et al., 2003) to manipulation methylation in newborn pups. We found that zebularine treatment increased RSC-V1aR in LO/LO 21d animals but not in their HI/HI siblings (Okhovat et al., 2017a, Figure 4C). Overall, these data present a remarkably coherent picture in which the high CpG density of LO alleles made them both more sensitive to the silencing effects of the oxytocin receptor antagonist, and to the demethylating effects of zebularine. LO alleles seem to be more developmentally sensitive, while HI alleles seem to be constitutively highly expressing.

While HI and LO alleles differ in their sensitivity to developmental perturbation, examination of the methylation of the intron enhancer suggests a more complex story than we hypothesized. Enhancer methylation *was not* influenced by these developmental manipulations (Okhovat et al., 2017a). While HI and LO genotypes exhibited GxE interactions, this effect does not seem to be due to CpG density differences in the putative intron enhancer alone. It is likely that genetic differences in the intron enhancer are inherited along with genetic variation at additional unexamined enhancers. Indeed, methyl-DNA immunoprecipitation (meDIP) identifies additional differentially methylated near *avpr1a*, but outside of our original focus (Okhovat et al., 2017a, Figure 5). Examining whether any of these sites also contain sequence differences between HI and LO alleles may clarify how genetic variation in *avpr1a* regulatory mechanisms contributes to sensitivity to developmental perturbation, and how these interact with regulatory regions we have already identified.

**Figure 5:**
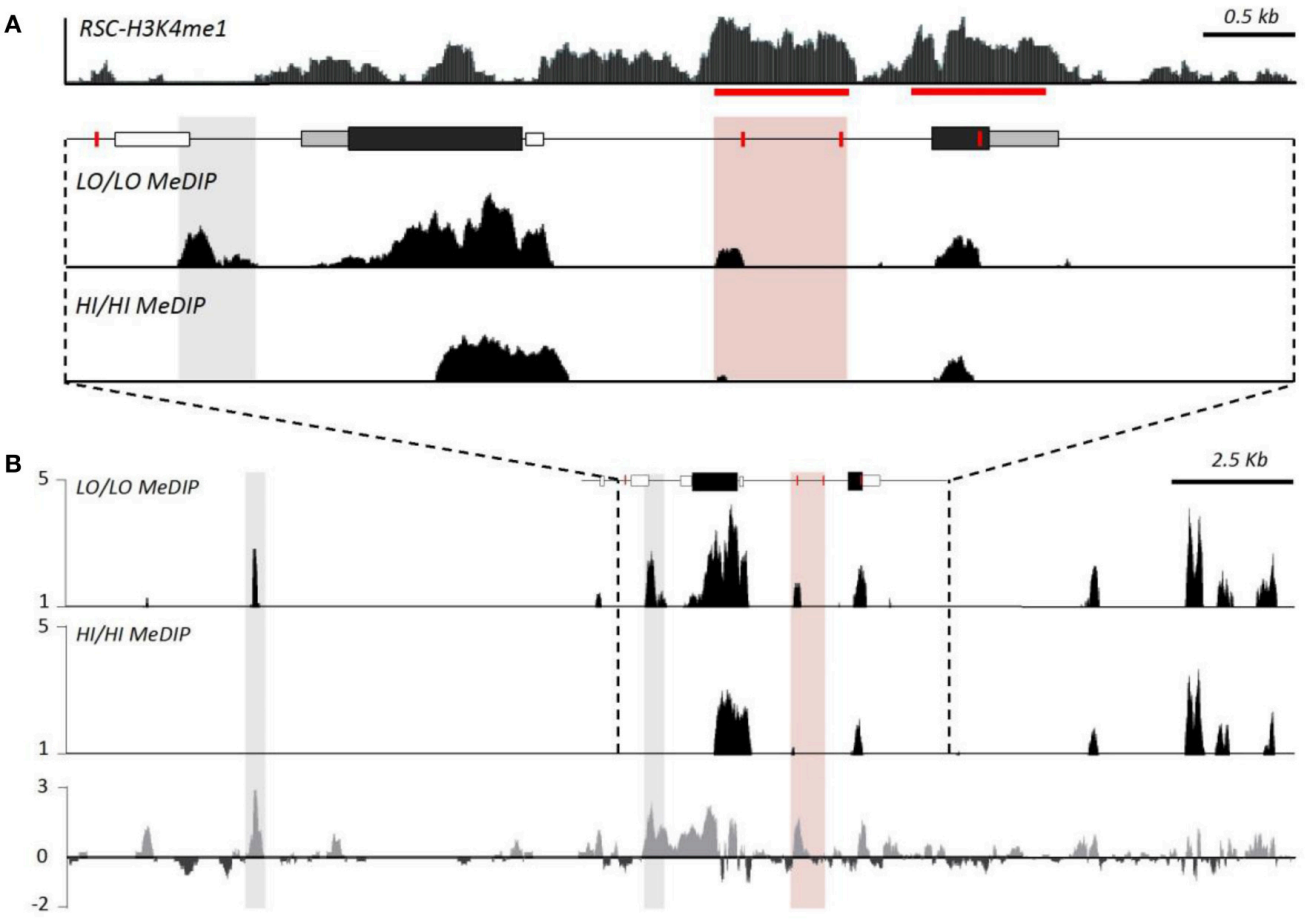
Genotype differences in methylation suggest distal regulators of avpr1a function. **(A)** Top panel depicts relative read depth (fold enrichment) of H3K4me1 reads denoting putative enhancers at the avpr1a locus. Bottom panels depict read depths from methyl-DNA immunoprecipitation sequencing (meDIP) targeting the RSC of HI/HI and LO/LO prairie voles. The data confirm enhanced methylation near SNP 2170 (pink box) of LO/LO genotypes, as well as revealed a differentially methylated region (DMR) just upstream of the transcription start site (where there are no genetic differences between HI and LO alleles). **(B)** A more expanded view of the locus reveals a strong DMR ~10 kb upstream of the locus; it is not known whether this or other more distal sites also differ in their underlying sequence. Modified with permission from Okhovat et al. (2017b).

## Conclusions and future directions

Our work began with the observation that the distribution of vasopressin 1a receptor in the RSC was surprisingly variable across individuals (Insel et al., 1994; Phelps and Young, 2003). We found that this variation predicted patterns of space-use and sexual fidelity in the field, with high levels of RSC-V1aR associated with sexual fidelity, and low levels associated with infidelity—even among paired males (Ophir et al., 2008c; Okhovat et al., 2015). Field paternity data and patterns of standing variation within the genome both suggest that variation at the *avpr1a* locus has been actively maintained by selection. Lastly, the alleles that drive differences in RSC-V1aR influence not only the mean level of vasopressin receptor, but also its sensitivity to developmental perturbation. Although this work spans diverse levels of analysis, from the function of chromatin to tests of selection in natural environments, there are a number of interesting questions that remain unanswered.

From a molecular perspective, while HI and LO alleles cause differences in RSC-V1aR abundance, we do not yet understand how nucleotide variation translates into differences in *avpr1a* function. Which of the four linked SNPs, if any, are causal? The case is strongest for the intron SNP 2170: it is a polymorphic CpG site associated with a cluster of polymorphic CpGs; it occurs within a region of chromatin that displays an enhancer-specific histone mark; it exhibits differential methylation between genotypes; its methylation status is associated with RSC-V1aR in animals from both lab and field; and it exhibits a pattern of nucleotide diversity that indicates a history of balancing selection (Okhovat et al., 2015, 2017a,b; Berrío Escobar, 2017). The 5′ SNP (-1396) has been less studied, but is also promising. It flanks a strongly conserved CTCF binding site and resides within a region of open chromatin (Okhovat et al., 2015, Figure 1A). The unusually tight linkage between these sites similarly suggests some coordinated function (Berrío Escobar, 2017). These data, however, fall short of demonstrating that either of these SNPs is causal. Moreover, the fact that developmental perturbations influence RSC-V1aR without altering the methylation status of the intron enhancer suggest that there are other, more distal regulators—an interpretation reinforced by the existence of differentially methylated regions outside of the immediate *avpr1a* locus (Figure 5). Whether such distal regulators bear sequence variation that contributes to HI and LO alleles remains to be determined. Our ChIP-seq approach allows for the exhaustive identification of distal regulatory sites, but conformation capture methods such as Hi-C will be needed to identify sites that make contact with *avpr1a* promoter, and that are thus likely to be directly shaping *avpr1a* function (Mifsud et al., 2015). Gene therapy methods using *cas9* to target deletions of putative enhancers, or using inactivated *cas9* fused to chromatin-remodeling enzymes to shape the function of specific regulatory sequences (Senís et al., 2014) provide a means for more directly determining whether specific nucleotides shape cortical expression of the *avpr1a* locus, and how such nucleotides interact with developmental experience.

While the molecular underpinnings of RSC-V1aR will offer novel insights into the nature of GxE and their substrates, a second series of unanswered questions concerns the exact nature of the relationship between RSC-V1aR and behavior. The behavioral functions of the RSC are an area of active investigation in both humans and traditional laboratory rodents. From a neuroanatomical perspective, the RSC is interconnected with the hippocampus, entorhinal cortex, anterior thalamus, and laterodorsal thalamus—a circuit central to episodic and spatial memory (Aggleton, 2014). Indeed, the RSC is active during navigation tasks, and in rats the RSC contains head-direction cells (Vann et al., 2009; Todd and Bucci, 2015). Imaging studies of humans (and rodents) at rest reveal that the RSC is one of two major nodes of the “default mode network”—a group of brain regions active when not performing a task (Spreng et al., 2008; Lu et al., 2012; Stafford et al., 2014). The second major node is the anterior cingulate cortex, a major target of the RSC (Spreng et al., 2008; Lu et al., 2012; Stafford et al., 2014). One interpretation is that the RSC connects a posterior circuit that processes memory, with a more anterior prefrontal circuit that processes decision-making; in human studies, the default mode network activity is sometimes interpreted as daydreaming, in which memory is used to simulate possible actions (Spreng et al., 2008).

Causal manipulations of RSC function confirm its role in a variety of memory-related tasks, but there is not a clear consensus on exactly how the RSC contributes to memory. In one recent study, Cowansage et al. (2014) used activity-dependent expression of channel rhodospins to tag and manipulate RSC neurons that were active during exposure to a shock-associated context. They found that activation of these neurons could elicit freezing responses in the absence of the context. One interpretation of these data is that the RSC serves to either encode or retrieve long-term memories and, through its reciprocal projections with the hippocampus, allow access to those memories during related experiences (Todd and Bucci, 2015).

The existing literature suggests a variety of alternative hypotheses for the role of RSC-V1aR in space-use and sexual fidelity. Our core observation is that a male with high V1aR intrudes less on territories of neighboring males, more effectively guards his mate, and mates predominantly with his partner. One hypothesis is that animals with high V1aR are better able remember the locations of social interactions—this could translate into the observed patterns of space-use and fidelity by making high V1aR males better able to guard mates (Okhovat et al., 2015). Similarly, having low cortical V1aR may impair the ability to recall locations of punitive encounters, making low-V1aR males more likely to intrude on neighboring territories and gain extra-pair copulations (Ophir et al., 2008c). In addition, there may be something non-spatial about the role of the RSC in social interaction—it may shape memory for one's partner, for example, or facilitate discrimination between remembered individuals through its projections to prefrontal cortices. Whatever the pattern proves to be, a rich set of studies aimed at dissecting the cognitive aspects of bonding, navigation, choice, and fidelity remain to be done.

Aside from the specific insights the above studies offer, they also provide a general framework for thinking about variation in the nervous system and its relationship to social behavior. First, they demonstrate that genetic variation in brain function can be a source of adaptive behavioral variation within a species. Our understanding of genetic variation in the nervous system is incredibly understudied, and this work provides a novel perspective on how diverse brains can be. A second value is that the studies illustrate how modern tools for interrogating chromatin function can be used to identify specific DNA sequences likely to be important to the regulation of behavior. On a genome scale, combining these sequencing tools with evolutionary genetics will allow researchers to more quickly identify which among the many thousands of regulatory sequences (and billions of nucleotides) are likely to be playing a causal role in gene expression (e.g., Pollard et al., 2006; Boyd et al., 2015). Lastly, we show how identifying nucleotide variation within specific regulatory sequences allows one to explore the interactions between genetic and epigenetic variation. Together such approaches will be a tremendous aid not only to our understanding of natural behavior, but in our quest to identify how variation in the genome interacts with the environment to shape the diversity of social behavior related to both health and disease.

## Author contributions

SP conceived and outlined the review, wrote the introduction, conclusion and future directions, assigned manuscript tasks and revised all other sections. MO wrote the sections on genetics and epigenetics. AB wrote the section on evolutionary analyses.

### Conflict of interest statement

The authors declare that the research was conducted in the absence of any commercial or financial relationships that could be construed as a potential conflict of interest.
